# En route to precision medicine for mental health: World Mental Health Day 2021

**DOI:** 10.1038/s42003-021-02681-2

**Published:** 2021-10-08

## Abstract

October 10^th^ marks World Mental Health Day. In an increasingly uncertain world, an understanding of how we can manage our own mental wellbeing and treat mental health conditions is more important than ever. Although much progress has been made in recent years in terms of our abilities to both diagnose and treat mental health conditions, the need to advance our understanding of the underlying mechanisms is continuous. In honour of World Mental Health Day this year, *Communications Biology* has gathered a Collection of our publications that fill some of the gaps in our current knowledge.

The world has come a long way in how we understand and treat mental health conditions. Although it is impossible to pinpoint the exact ‘discovery’ of depression, some of the first accounts include those written as early as the second millennium B.C.E. in Mesopotamia. However, when first discussed, depression was considered to be a spiritual phenomenon rather than medical and as such was preferentially treated by priests rather than doctors^1^. It was Hippocrates who suggested that depression, or ‘melancholia’, was instead caused by ‘an imbalance of bodily fluids’^2^ and thus begins our long journey to what we understand about depression and other mental health disorders today.

The theme of this year’s Mental Health Day is ‘Mental Health in an Unequal World’. We hope that speaking about mental health and sharing the science as widely as possible will play a small part in addressing the inequities faced globally in terms of mental health care. Despite the advances that we have made, both scientifically and as a society, there is still a regrettable stigma surrounding mental health conditions. Our understanding about the underlying mechanisms and the precise effects of therapies is still very much incomplete. *Communications Biology* is committed to not only breaking down the stigma that is still associated with mental health conditions, but is also dedicated to publishing high quality science that builds upon our understanding of all mental health disorders. Within our pages, we are grateful to have some intriguing papers that have expanded our understanding of the underlying mechanisms of anxiety and trauma both in mouse models and in humans. Animal models are invaluable in the hunt to pinpoint precise, disorder-specific mechanisms. However there are always likely to be some limitations with respect to how much translational value they have. One key approach is to carry out cross-species assessments, and that is exactly what Benzina et al. have recently done in the context of compulsive behaviour^3^. In doing so, they provide crucial information that helps to optimize the animal models used in this context. We strongly encourage submissions that take a similar approach.

Speaking about mental health and sharing the science as widely as possible will play a small part in addressing the inequities faced globally in terms of mental health care.

Despite the wealth of information that animal models have given us, one of the main issues with understanding mental health disorders is how heterogenous they are between different people. There is no one single cause or one silver bullet to treat all symptoms. The way forward is to steer research towards developing personalised medicine and take into account all players, including genetic risk factors and environmental factors. We very recently published a fantastic paper from Hunjan et al. in which they examined the association between polygenic propensity for mental health disorders and nutrient intake^4^. This begins to pave the way for the identification of patient-specific underlying mechanisms and therefore more targeted treatment for disorders such as Anorexia Nervosa. Not only can a more personalised approach help us to develop more innovative treatments, but it can also help us make more reliable and efficient diagnoses, which is very much needed for optimum mental health care as many individuals may wait years for a final diagnosis. Another hurdle faced by many with mental health disorders is the multitude of distressing side-effects that are caused by subscribed medication and in some cases, the side-effects result in therapy withdrawal. Precision medicine can help us predict how effective and how well-tolerated medications will be.

Although personalised medicine will be indescribably invaluable, we must also consider the huge effect of the world around us, both in terms of stressful events and our environment. In an ever changing society, many lean on alcohol or other substances to soften the blows that life deals. Indeed, addiction can take many forms and all have a negative impact on our mental health. As an example, with an ever-increasing number of people spending more and more time working and living virtually, online gaming addiction is coming into the forefront. A better understanding of the effects of addiction and how our brains change as we become addicted to substances or activities will inevitably help us tackle this burden.

One thing that comes hand in hand with many addictions, as well as the current pandemic, is lack of sleep. The relationship between sleep and mental health is so bidirectionally intertwined that one cannot consider mental health disorders without thinking about sleep. Sleep is so crucial that improving its quality and duration is probably the most consistent goal for anyone with a mental health disorder and indeed even for those who don’t have one. Research on the biological mechanisms underlying sleep and their relationship to overall health is just as necessary as psychological research. For example, a recent study from Hughes et al^5^. focused on the role of timed exercise in sleep and in how it specifically affects the circadian rhythms of mice at a cellular level. While we have appreciated for a while now that exercise is beneficial for improving our sleep, this study helps us to begin to understand exactly why.

The efforts towards a better understanding of the mechanisms underlying mental health disorders and therefore an improvement in diagnosis and therapeutic interventions are ongoing. *Communications Biology* will continue to encourage the publication of science that helps us to achieve this goal. Some of our existing publications have been highlighted here but we have many others that contribute to our understanding of mental health disorders and we have created a Collection to showcase them here. We proudly join the global effort to shine a spotlight on mental health and the science that will drive improvements in mental health care and society’s perception of mental health disorders.
